# Supplementary material for: A multicenter longitudinal study of cholinergic subgroups in Parkinson disease
Source: Nat Commun. 2025 Jul 1;16:5655. doi: 10.1038/s41467-025-60815-0 (PMC12219741; doi:10.1038/s41467-025-60815-0)
Supplement: Supplementary file 1 — Supplementary Information [file 41467_2025_60815_MOESM1_ESM.pdf]

Supplementary Materials

Section 1: Group comparisons of PD patients at baseline by site against pooled sample of controls

Pooled sample of control participants (N=31) had a mean age of 67.1 [64.1, 70.0] years, with no statistically significant differences between PD patients in either site (University of Michigan [UofM] or University of Groningen [UofG]) relative to controls on age (F=0.409, p=0.665). Controls had a mean MoCA score of 27.7 [26.6, 28.8] points, with statistically significant differences relative to PD patients (F=10.42, p<0.001), both among UofM patients (B=-1.51 [-2.69, -0.33] points, p=0.0123) and UofG patients (B=-2.71 [-3.94, -1.48] points, p<0.001). The distribution of sex was unequal between PD patients in both sites relative to controls (*Fisher's exact test p-value:* 0.004), with 45% of the pooled control sample being male, whereas 75.8% and 67.7% of the patient samples were male in UofM and UofG respectively. These findings suggest that our PD patients were matches on age relative to controls, had poorer cognition, and tended to have a greater proportion of males.

Section 2: Longitudinal sample group comparisons between cohorts

Table S1. Mean (± SD) values of follow-up sample demographic and clinical variables of the combined Michigan and Groningen group, individual center values, and statistical comparisons between the two groups. Gender distribution is presented as proportions. Unequal variance independent samples *t*-tests were applied to test group differences in numerical variables while chi-square contingency tests were used for categorical variables. Statistically significant two-tailed comparisons at uncorrected α=0.05 are marked with an asterisk.

Variable	*Combined (N=128)*	*Michigan (N=72)*	*Groningen (N=56)*	*Statistic*	*P*
Sex male/female (Male % total)	94/34 (73.4%)	56/16 (77.8%)	38/18 (67.8%)	χ^2=1.121	0.2896
Age (years)	68.9±7.3	68.61±6.6	69.18±8.08	t=-0.426	0.6707
MoCA score	26.2±3.1	26.91±2.48	25.2±3.63	t=3.024	0.0032*
MDS-UPDRSII total score	9±5.8	7.93±5.2	10.48±6.3	t=-2.449	0.016*
Hoehn & Yahr	2.1±0.6	2.42±0.52	1.79±0.59	t=6.271	<0.001*
Motor disease duration (years)	6.2±3.2	7.42±3.81	4.62±0.84	t=6.037	<0.001*
LED (mg)	679.9±413.5	735.92±470.9	607.79±314.83	t=1.84	0.0682
Years since baseline	2.5±0.6	2.12±0.53	3.06±0.1	t=-14.685	<0.001*

Section 3: Imaging Methods

Michigan: Magnetic resonance imaging (MRI) was performed on a 3 Tesla Philips Achieva system (Philips, Best, The Netherlands). A 3D inversion recovery-prepared turbo-field-echo was performed in the sagittal plane using TR/TE/TI=9.8/4.6/1041ms; turbo factor=200; single average; FOV=240x200x160mm; acquired Matrix = 240x200x160 slices and reconstructed to 1mm isotropic resolution. PET imaging was performed in 3D imaging mode with a Siemens ECAT Exact HR+ tomograph or Biograph 6 TruPoint PET/CT scanner (Siemens Molecular Imaging, Inc., Knoxville, TN), which acquired 63 transaxial slices (slice thickness: 2.4 mm) over a 15.2 cm axial field-of-view (HR+) or 109 transaxial slices (slice thickness: 2.028 mm) over a 22.0 cm axial field-of view (BioGraph). To further minimize the impact of using two different scanners, an inter-scanner data harmonization was performed (*1*). Images were corrected for scatter and motion. Subjects on dopaminergic medications were scanned in the dopaminergic medication 'on' state.

Groningen: Brain MRI was performed using Siemens Magnetom Prisma 3 T MRI scanners, equipped with SENSE-8 channel head coil. For each subject, anatomical T_1-weighted images were obtained using a sagittal 3D gradient-echo T_1-weighted sequence with 0.9 × 0.9 × 0.9 mm3 acquisition. Control subjects underwent a T_1-weighted MRI scan (3 T Intera) with 1.0 × 1.0 × 1.0 mm^3 acquisition. PET was performed on the same day as MRI. For $[^{18}F]$-FEOBV PET imaging, participants first underwent lose-dose CT for attenuation and scatter correction using either a Biograph 40-mCT or 64-mCT (Siemens Healthcare). Both scanners were EARL certified, had the same software version and used identical acquisition and reconstruction protocols and PET detectors.

$[^{18}F]$FEOBV was prepared as described previously (*2*, *3*). $[^{18}F]$FEOBV delayed dynamic imaging was performed over 30 minutes (in six 5-minute frames) starting 3 hours after an intravenous bolus dose injection of 8 mCi $[^{18}F]$-FEOBV (*4*) at the two centers. All Groningen data were reconstructed following the identical PET processing protocol used at the University of Michigan. All PET image postprocessing, including distribution volume ratio (DVR) calculations and parameterization for voxel-based analysis, was performed at the University of Michigan. The PET imaging frames were spatially coregistered within subjects with a rigid-body transformation to the first frame to reduce the effects of subject motion during the imaging session (*5*). ANTsPyX open source *Python* programming language module was used for PET-MRI registration. A supratentorial white matter reference tissue approach was used to determine VAChT binding as previously reported (*6*, *7*). A FreeSurfer segmentation was used to obtain a mask of all cerebral white matter voxels lying above the lateral ventricle, which was subsequently eroded with a 3mm radius sphere and moved to the PET image space using the inverse PET-MRI rigid body transformation. Distribution volume ratios (DVR) were calculated from a ratio of the average of six delayed imaging frames (3 hours after injection) for gray matter target and supratentorial white matter reference tissues (*7*). For the voxelwise analysis, all brain images were spatially normalized to Montreal Neurological Institute (MNI) template space using DARTEL normalization protocol and smoothed with a Gaussian kernel of 8 mm full width half maximum to adjust the anatomical variability between the individual brains and to enhance the signal-to-noise ratio. Before normalizing and smoothing the parametric PET images into MNI, the images were corrected for partial volume effect using the Müller-Gärtner method as previously described (*8*).

Section 4: System-level cholinergic principal components

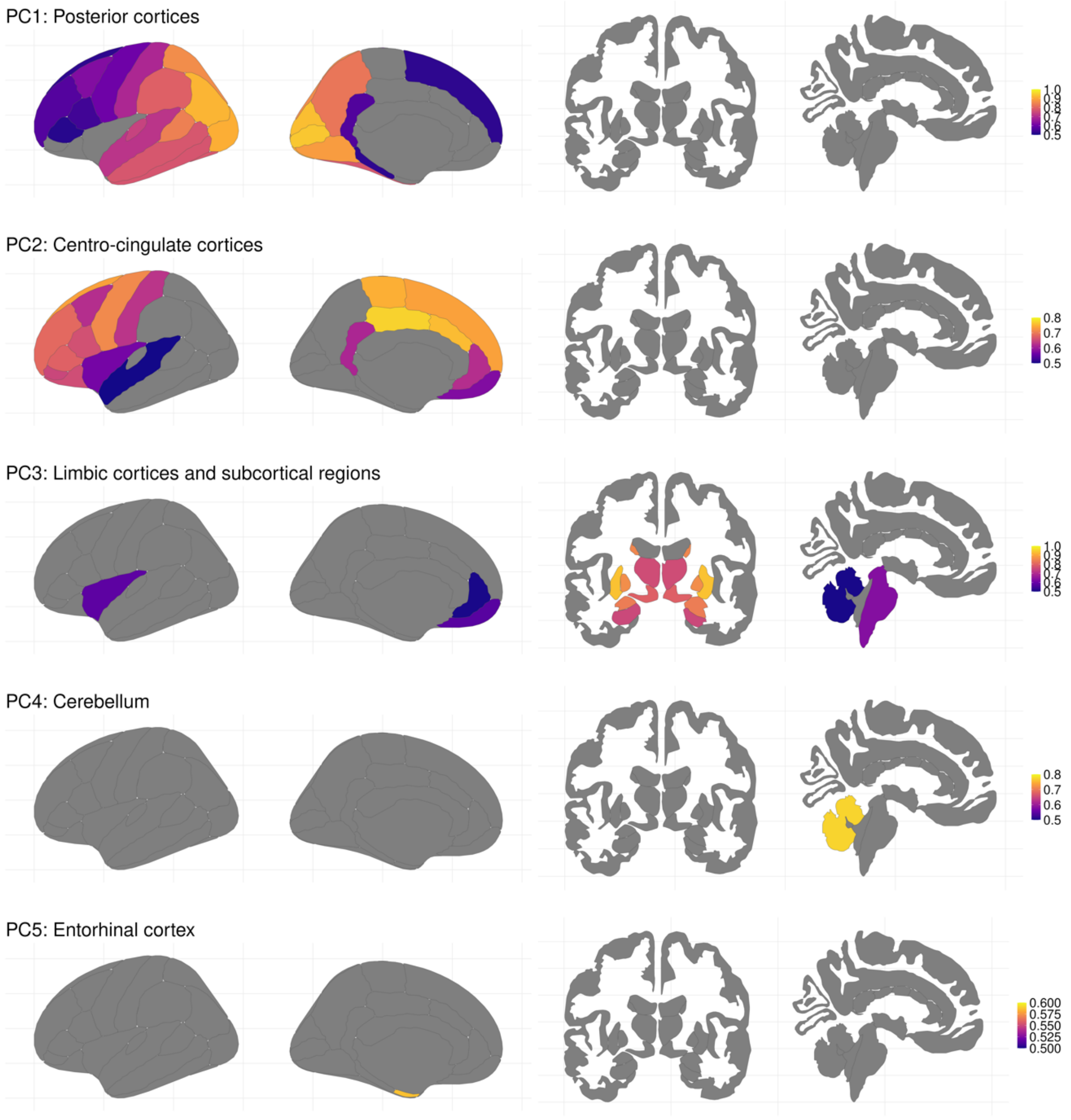

Figure S1. Principal component analysis varimax-rotated and thresholded loading scores, visualized on top of corresponding cortical/subcortical brain regions. Colors closer to blue specify weaker loadings, colors closer to yellow specify stronger loadings unto the principal component.

Section 5: Reference region post-hoc validation

To ensure that our choice of reference region was suitable for comparing the systems-level cholinergic subgroups against controls and for evaluating within-subject interval changes, a post-hoc mixed linear model analysis was performed on supra-ventricular reference region

mean standardized uptake values (SUV). SUV represents a commonly used semi-quantitative measure of radiotracer uptake, expressed as a ratio of radioactivity (in becquerels per milliliter; *Bq*/*mL*) observed in a region of interest over a theoretical quantity which would be expected assuming that the injected radiotracer dose (in *Bq*) would distributed uniformly throughout the patient's body volume (in milliliters [mL], estimated from patient's weight in grams [g] and assuming body density of 1 g/mL). SUV allows for comparison of radiotracer uptakes while accounting for potential inter-subject variability in observed signal that is related to the injected dose and body volume. Two participants from University of Groningen were not included in the present analysis because their weights were not recorded during the clinical visit, thus precluding us from computing their reference region SUV. Mean SUVs were compared by subgroup (HYPER, MIXED, HYPO vs. controls) and visit (follow-up vs. baseline) to ensure that no systematic differences are observed in reference region signal that would directly affect these further analyses by systematically biasing our quantification of brain VAChT distribution. Furthermore, to demonstrate that no systematic influence on our reference region quantification approach was imposed by heterogeneity in site (Groningen vs. Michigan), scanner (HR+ vs. BioGraph), and age within the combined multi-centric dataset, these variables were included as covariates in the model.

Table S2. Mixed linear model comparing reference region SUV of each PD system-level cholinergic subgroup against controls and within-subject longitudinally. Statistically significant model coefficients at uncorrected two-tailed α=0.05 are marked with an asterisk.

		**SUV (Z)**	
Predictors	*Estimates*	*CI*	*p*
Normal Controls (Intercept)	-0.145	-0.551 – 0.261	0.483
Subgroup [HYPER]	0.002	-0.449 – 0.453	0.992
Subgroup [MIXED]	0.172	-0.253 – 0.598	0.427
Subgroup [HYPO]	0.309	-0.118 – 0.736	0.155
Site [GRON vs. UMICH]	-0.124	-0.403 – 0.155	0.384
Age (years)	0.127	0.005 – 0.250	0.042*
Visit [Follow-up vs. Baseline]	0.078	-0.038 – 0.193	0.186
Scanner [HR+]	0.183	-0.015 – 0.382	0.070
**Random Effects**			
σ^2	0.15		
$\tau_{00\ sub}$	1.00		
ICC	0.87		
N_{sub}	274		
Observations	402		
Marginal R^2 / Conditional R^2	0.046 / 0.873		

The obtained mixed linear model coefficient estimates presented in Table S2 suggest that no statistically significant difference was observed between any of the PD cholinergic subgroups and our sample of controls. The non-significant estimate for the hyper-cholinergic group was very close to 0, suggesting minimal if any influence of reference region related quantification bias. The directionality of the non-significant coefficients for the mixed- and hypo-cholinergic subgroup trended in the positive direction, suggesting that if there is any reference region related quantification bias, it would lead to potential over-estimation of down-regulation relative to controls and under-estimation of up-regulation relative to controls. These findings support the conjecture that our chosen reference region based quantification approach is appropriate for comparing against normative controls (no evidence of subgroup-specific quantification bias). The within-subject effect of visit (follow-up vs. baseline) was also not statistically significant, which supports the conjecture that our reference region based quantification approach is appropriate for evaluating within-subject interval changes. Graphical evidence to support these conclusions is presented in figure S2. While no confounding influence of site was observed on the reference region SUV, there was a marginally significant trend for confounding influence of heterogeneity in scanner and participant age. Due to these findings, scanner and age were included as covariates in subsequent analyses as appropriate.

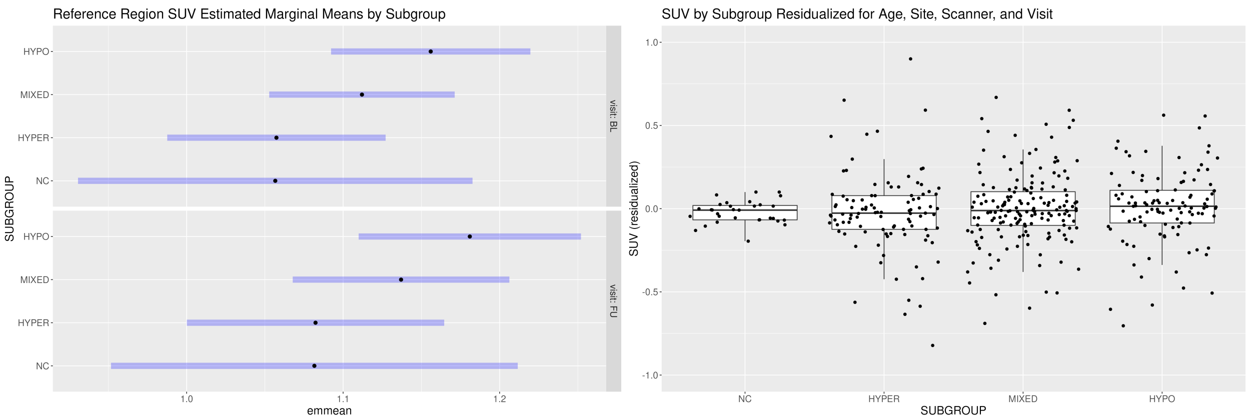

Figure S2. Estimated marginal means plot (on the left) shows the 95% confidence intervals for the subgroup mean SUV value at baseline (on top left) and follow-up (on bottom left) visits. In all cases, mean estimates for the cholinergic PD subgroups fall within the 95% confidence intervals of controls. Overall, SUV values are very close to 1 in all subgroups, suggesting that radiotracer distribution in supra-ventricular white matter is very low, which supports its use as a reference region for quantification. On the right, a box and jitter plot of reference region values by subgroup is shown after covarying out the influence of age, site, scanner, and visit. While PD subgroups appear to have greater variability in reference region SUV, the median estimates on the boxplots for the subgroups essentially overlap with the median estimate for controls, which supports the conjecture that no subgroup-specific quantification bias is at play in the present dataset.

Section 6: Post-hoc analysis of structural brain atrophy in combined sample of PD patients and by cholinergic subgroup relative to controls

To support our use of PVC in subsequent voxelwise analyses, a post-hoc analysis was performed examining whether the combined sample of PD patients (N_{PD}=245) and individual PD cholinergic subgroups (N_{HYPER}=71, N_{MIXED}=100, N_{HYPO}=74) exhibited appreciable evidence of structural brain atrophy relative to controls (N_{NC}=31). Two measures of structural brain atrophy were utilized: total gray matter volume normalized by intracranial volume and the discrepancy between estimated brain age and the participant's biological age. Regional gray matter volumes were obtained using *FreeSurfer*, summed, and divided by total intracranial volume to obtain normalized gray matter volume (lower values indicate more severe atrophy, age was included as covariate in the group comparison). Brain age was estimated using the *brain_age* function implemented by *ANTsPyNet* (*9*), and biological age was subtracted from brain age estimate to obtain the discrepancy between brain age and biological age (higher values indicate more severe atrophy than would be expected due to age alone).

The combined sample of PD patients did not differ significantly relative to controls on normalized gray matter volume after adjusting for age (β=0.13 [-0.17, 0.43], p=0.391) or brain age discrepancy (β=0.19 [-0.19, 0.56], p=0.332). Neither mixed-cholinergic (β=0.17 [-0.14, 0.49], p=0.272) nor hypo-cholinergic (β=-0.2 [-0.52, 0.13], p=0.235) PD patients differed significantly relative to controls on normalized gray matter volume after adjusting for age. Hyper-cholinergic PD patients were interestingly found to have higher normalized gray matter volume relative to controls after adjusting for age with a moderate standardized effect size (β=0.41 [0.08, 0.74], p=0.014). Neither hyper-cholinergic (β=-0.01 [-0.41, 0.4], p=0.978) nor mixed-cholinergic (β=-0.06 [-0.45, 0.32], p=0.747) different significantly with respect to brain age discrepancy with biological age relative to controls. Hypo-cholinergic subjects were found to have a significantly higher positive discrepancy between brain age and biological age relative to controls with a strong standardized effect size (β=0.7 [0.3, 1.1], p=0.001). Taken together, these findings suggest that the use of PVC in our sample is well supported, given the evidence of substantially higher brain atrophy among hypo-cholinergic PD patients as measured via brain age discrepancy with biological age. Higher normalized gray matter volume observed among hyper-cholinergic PD patients after adjusting for age relative to controls raise interesting questions related to factors which determine the extent of the hyper-cholinergic upregulation process which are however outside the scope of the present work.

Section 7: Voxel-level post-hoc cross-sectional comparisons of subgroups against controls by site with and without PVC

Post-hoc analysis limited to the Michigan data (N_{HYPER}=21, N_{MIXED}=68, N_{HYPO}=60) reproduced the overall topography of cholinergic upregulation in the hyper-cholinergic subgroup, though with notably attenuated upregulation observed in the hippocampi and amygdala, thalamus, and ventral striatum. While the mixed-cholinergic group findings in the Michigan-only post-hoc was largely comparable to the combined analysis, more extensive cholinergic losses were observed in the striata and thalami of hypo-cholinergic patients (figure S3).

--- CONTINUED ON NEXT PAGE ---

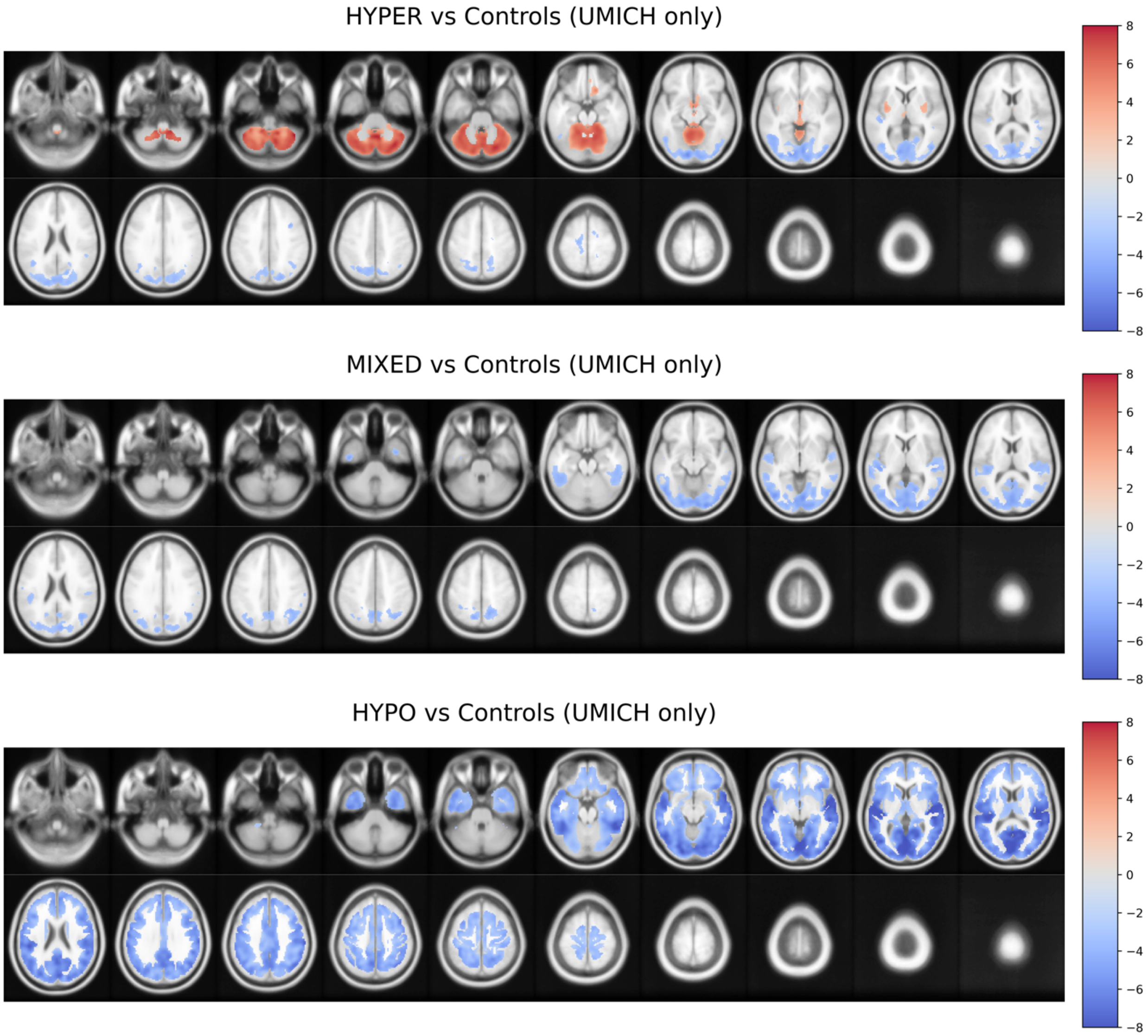

Fig. S3. Main voxel-based SPM analysis comparing each of the baseline cholinergic subgroups against control participants with independent samples t-tests (UMICH only). Statistical t-contrast images were thresholded with false discovery rate (FDR) threshold of $P<0.05$. Positive contrast values indicate statistically significant upregulation of [^{18}F]FEOBV uptake in a given region relative to control participants (colored in red), whereas negative values indicate statistically significant deficits relative to control participants (colored in blue).

Conversely, post-hoc re-analysis with Groningen-only cohort (N_{HYPER}=50, N_{MIXED}=32, N_{HYPO}=14) still demonstrated a comparable (though slightly more extensive) topography of upregulation among hyper-cholinergic patients, but with relatively attenuated posterior cholinergic deficits. Notably, Groningen mixed-cholinergic patients retained a partial topography of upregulation in the ventral striatum, putamen, mesencephalon, amygdala and hippocampus,

right basal forebrain, thalamus, and cerebellum, while exhibiting a largely comparable topography of downregulation. Lastly, the Groningen hypo-cholinergic subgroup still retained some upregulation relative to controls in the tectum and primarily cerebellar vermis, while also exhibiting relative sparing of cholinergic downregulation the striata, frontal cortices, thalami, limbic, and paralimbic regions (figure S4).

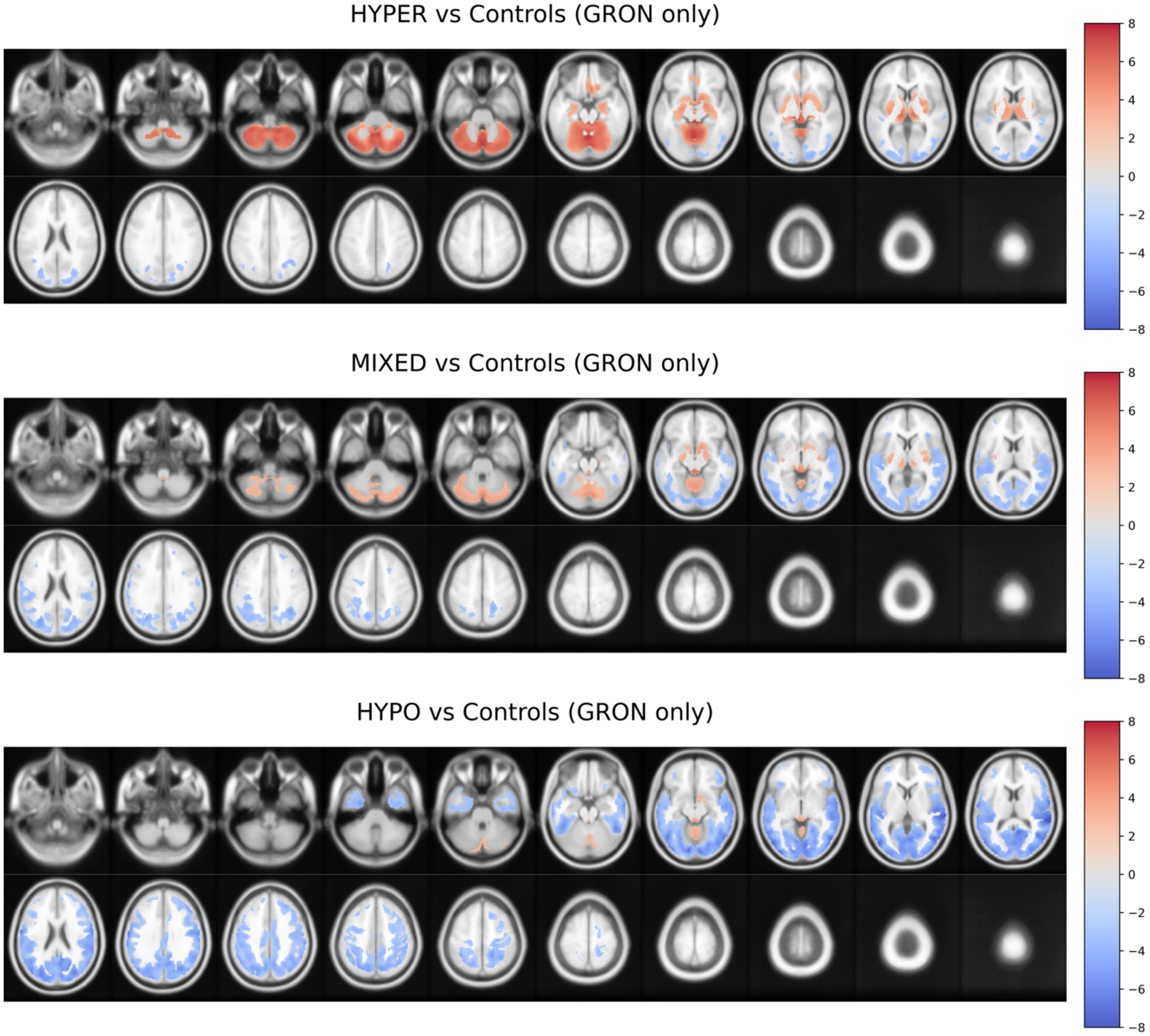

Fig. S4. Main voxel-based SPM analysis comparing each of the baseline cholinergic subgroups against control participants with independent samples t-tests (GRON only). Statistical t-contrast images were thresholded with false discovery rate (FDR) threshold of $P<0.05$. Positive contrast values indicate statistically significant upregulation of [^{18}F]FEOBV uptake in a given region relative to control participants (colored in red), whereas negative values indicate statistically significant deficits relative to control participants (colored in blue).

Analyses without PVC (figures S5-7) appeared to exhibit a wider overall spatial extent of statistically significant voxels, with more pronounced involvement of the centro-cingulate voxels in the upregulation topography (as compared to the original analyses with PVC). Retention of

upregulation in the cerebellar vermis in mixed-cholinergic subgroup was observed in the combined sample non-PVC comparison against controls, which agrees with the findings from the post-hoc Groningen only analysis presented above in figure S4.

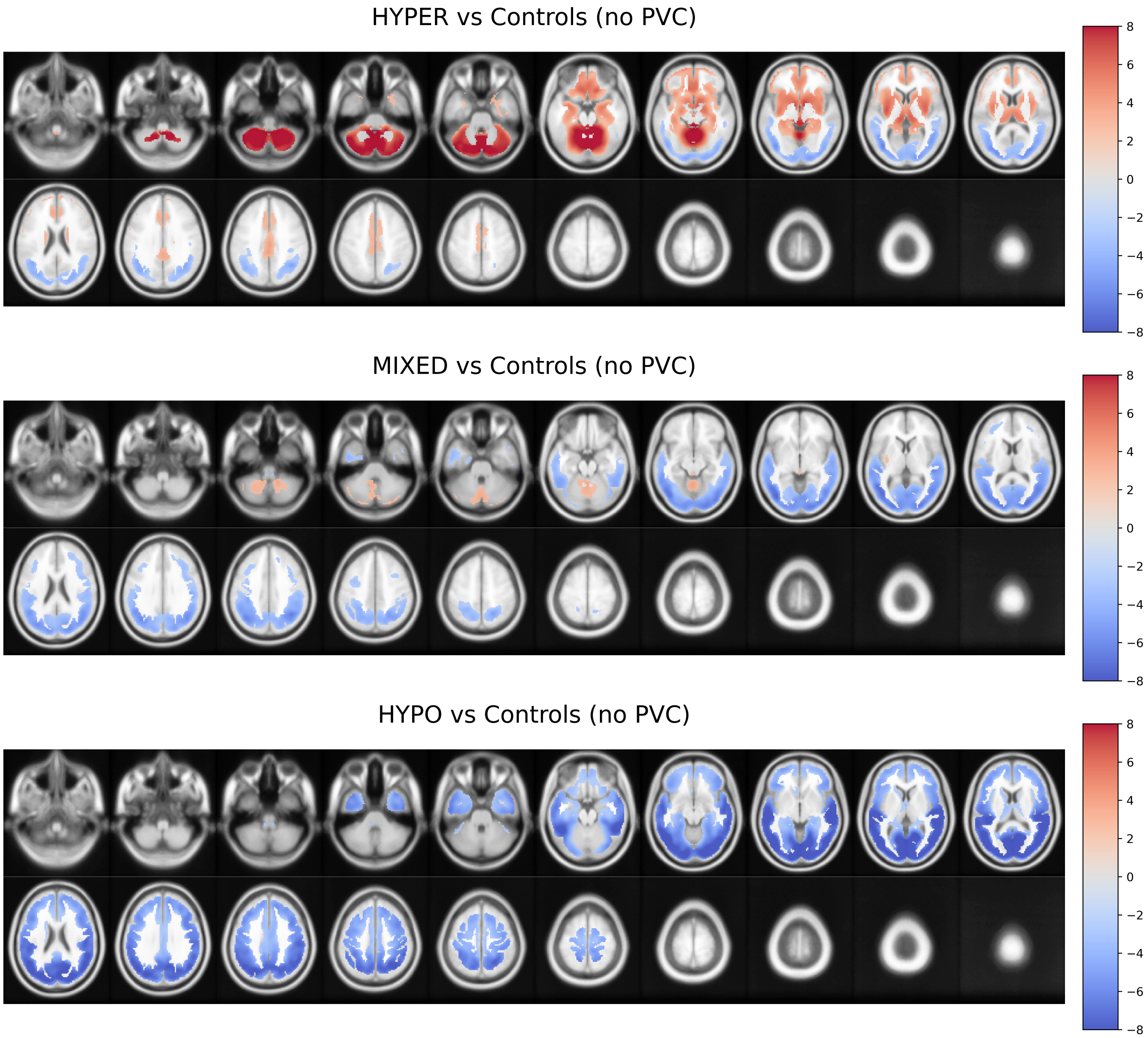

Fig. S5. Main voxel-based SPM analysis comparing each of the baseline cholinergic subgroups against control participants with independent samples t-tests (combined site, no PVC). Statistical t-contrast images were thresholded with false discovery rate (FDR) threshold of $P<0.05$. Positive contrast values indicate statistically significant upregulation of [^{18}F]FEOBV uptake in a given region relative to control participants (colored in red), whereas negative values indicate statistically significant deficits relative to control participants (colored in blue).

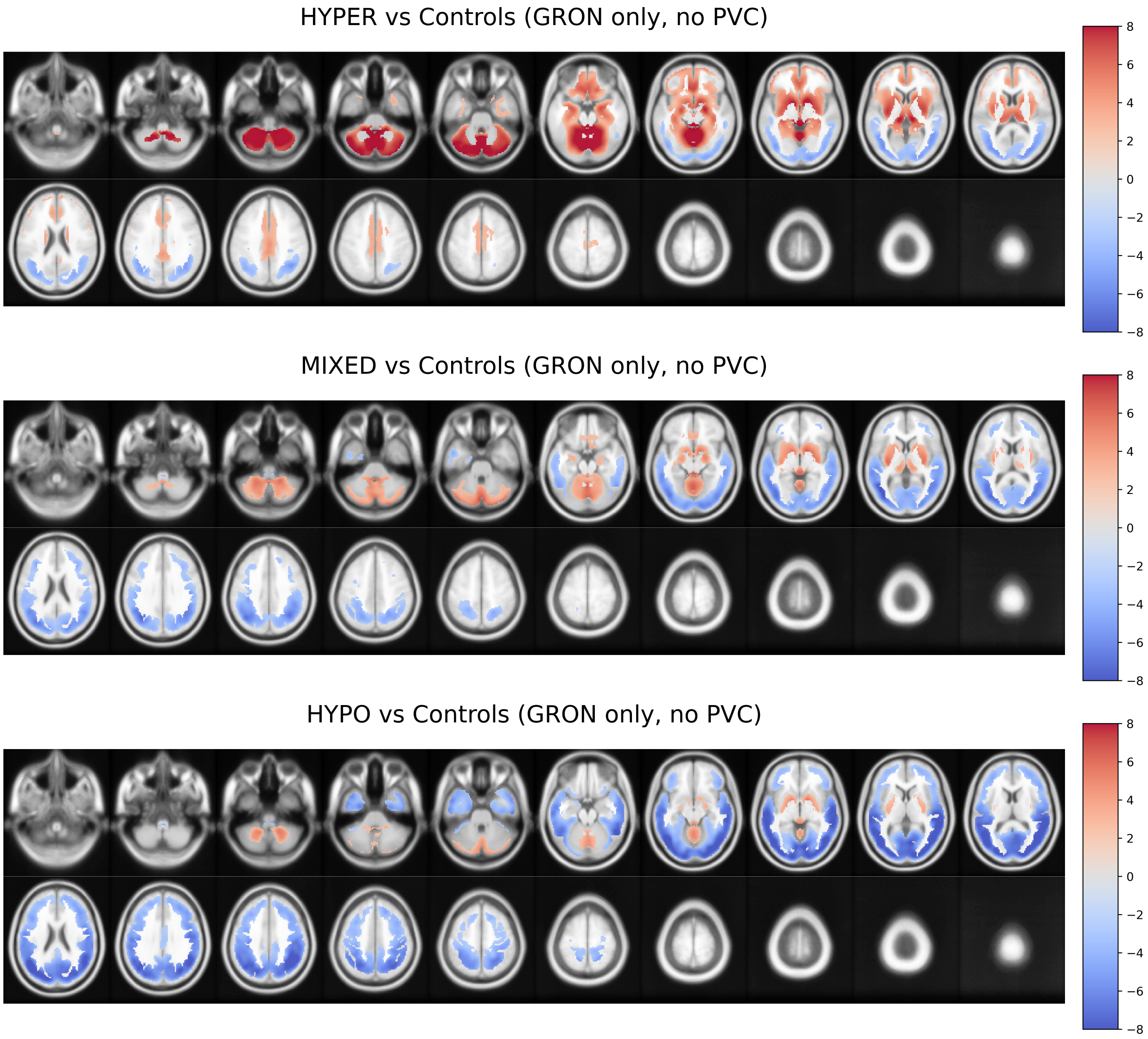

Fig. S6. Main voxel-based SPM analysis comparing each of the baseline cholinergic subgroups against control participants with independent samples t-tests (GRON only, no PVC). Statistical t-contrast images were thresholded with false discovery rate (FDR) threshold of P<0.05. Positive contrast values indicate statistically significant upregulation of [^{18}F]FEOBV uptake in a given region relative to control participants (colored in red), whereas negative values indicate statistically significant deficits relative to control participants (colored in blue).

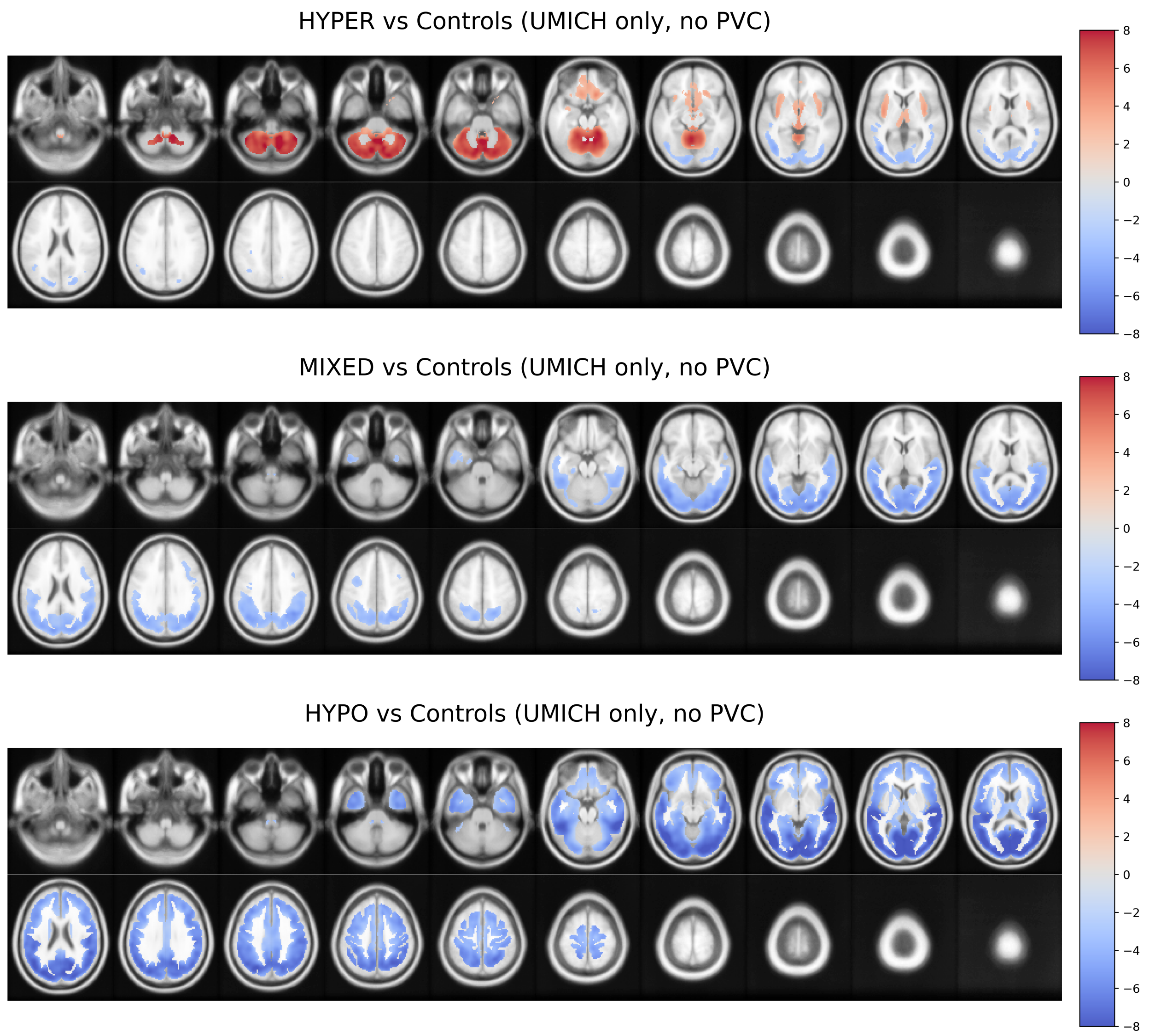

Fig. S7. Main voxel-based SPM analysis comparing each of the baseline cholinergic subgroups against control participants with independent samples t-tests (UMICH only, no PVC). Statistical t-contrast images were thresholded with false discovery rate (FDR) threshold of $P<0.05$. Positive contrast values indicate statistically significant upregulation of [^{18}F]FEOBV uptake in a given region relative to control participants (colored in red), whereas negative values indicate statistically significant deficits relative to control participants (colored in blue).

Section 8: Confirmatory systems-level principal component cross-sectional comparison against controls by subgroup.

To confirm that the voxel-level cross-sectional differences of the cholinergic subgroups against controls agree with the system-level picture, the subgroup comparison against controls was repeated using an *ANCOVA* group comparison, with adjustment for potentially confounding effects of age and scanner (see table S2). The hyper-cholinergic subgroup exhibited evidence of upregulation in the centro-cingulate, limbic/subcortical, and cerebellar principal components, with the latter two agreeing with the results of the main voxelwise group comparison, and the former agreeing with the post-hoc analysis without PVC as presented in figure S5. The mixed-cholinergic subgroup no longer shows evidence of upregulation in any of the components (in agreement with the primary voxelwise analysis) but exhibits more pronounced evidence of downregulation in the posterior cortical component (also in agreement with primary analysis). Lastly, the hypo-cholinergic subgroup exhibits both the most extensive decrease in cholinergic integrity across a range of components, with relative preservation of the cerebellar component. In conclusion, the system-level confirmatory analysis broadly agrees with the pattern of findings obtained with the more granular voxel-level primary analysis.

Table S3. Subgroup comparison against controls on system-level principal component scores. Model coefficients corresponding to group differences are presented with 95% confidence intervals. *F*-values and *P*-values for *ANOVA F*-test model comparison are presented. Strong regression coefficients (wherein the 95% interval does not overlap with 0) and statistically significant uncorrected two-tailed *P*-values (α=0.05) are marked with an asterisk.

Variable	*Hyper-cholinergic (N=71)*	*Mixed-cholinergic (N=100)*	*Hypo-cholinergic (N=74)*	*F*	*P*
PC1 (Posterior Cortices)	-0.125 [-0.434, 0.185]	-0.712* [-0.999, -0.426]	-1.63* [-1.921, -1.34]	73.142	<0.001*
PC2 (Centro-Cingulate)	+0.438* [0.107, 0.768]	-0.267 [-0.572, 0.039]	-1.22* [-1.53, -0.911]	63.215	<0.001*
PC3 (Limbic/Subcortical)	+1.086* [0.704, 1.469]	+0.098 [-0.256, 0.451]	-0.543* [-0.901, -0.184]	42.652	<0.001*
PC4 (Cerebellar)	+2.267* [1.771, 2.763]	+0.243 [-0.215, 0.702]	-0.446 [-0.911, 0.018]	76.644	<0.001*
PC5 (Entorhinal cortex)	+0.247 [-0.102, 0.596]	-0.178 [-0.501, 0.144]	-0.952* [-1.279, -0.625]	30.904	<0.001*

Section 9: Voxel-level within-subject longitudinal comparison of baseline PD cholinergic subgroups by site with and without PVC

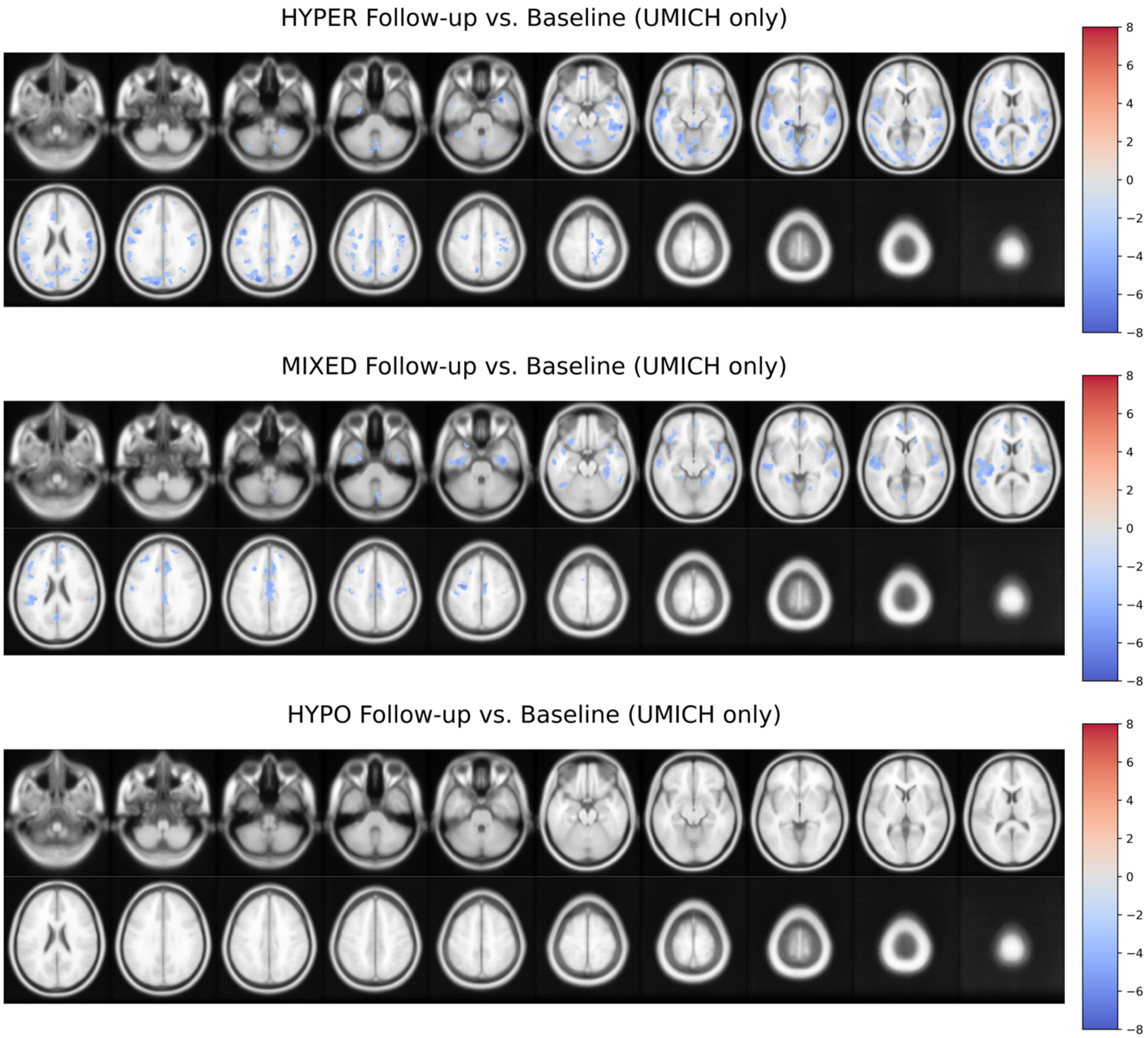

Fig. S8. Main voxel-based SPM analysis (UMICH only) comparing each subgroup within-subject at follow-up visits relative to baseline visits with paired samples t-tests (adjusted for days between visits, age, and scanner). Statistical contrast images (positive and negative) were thresholded with false discovery rate (FDR) correction at P<0.05. Positive contrast values indicate statistically significant upregulation of [^{18}F]FEOBV uptake in a given region relative to values observed at baseline (colored in red), whereas negative values indicate statistically significant deficits (colored in blue).

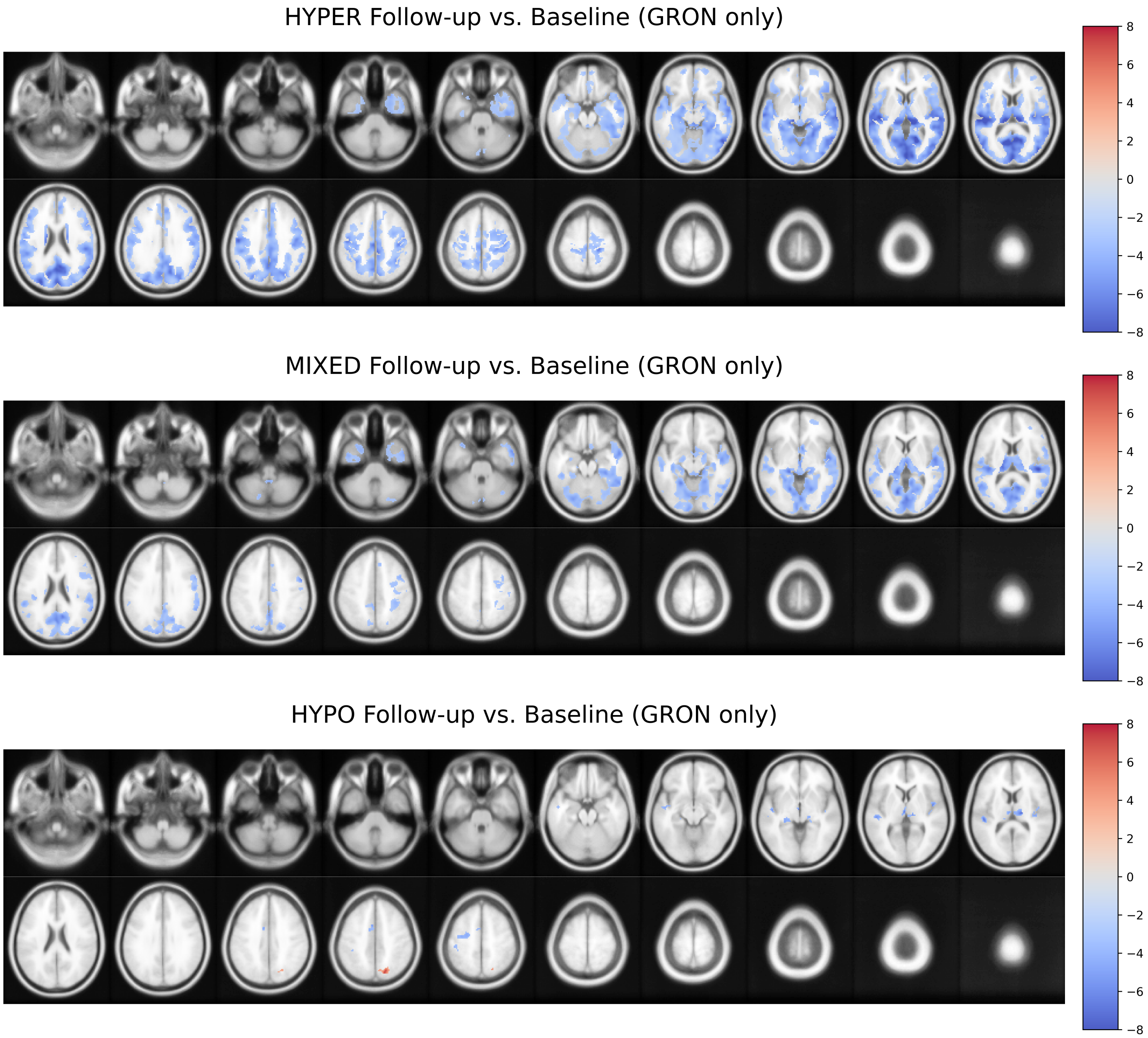

Fig. S9. Main voxel-based SPM analysis (GRON only) comparing each subgroup within-subject at follow-up visits relative to baseline visits with paired samples t-tests (adjusted for days between visits, age, and scanner). Statistical contrast images (positive and negative) were thresholded with false discovery rate (FDR) correction at P<0.05. Positive contrast values indicate statistically significant upregulation of [^{18}F]FEOBV uptake in a given region relative to values observed at baseline (colored in red), whereas negative values indicate statistically significant deficits (colored in blue).

Section 10: Global cholinergic system deviation summary measure integrative analysis with systems- and voxel-level

To capture a coarse-grained characterization of cholinergic system changes in PD, a set of whole-brain summary measures were defined from the parametric [^{18}F]FEOBV PET images spatially normalized to a standard template. The algorithm for obtaining counts of hyper-cholinergic and hypo-cholinergic voxels from which the global summary measures were derived is schematically represented in figure S10 and described in detail in methods subhead 6. Hyper-cholinergic voxel proportion (V_+) was defined to capture the global spatial extent of upregulation, hypo-cholinergic voxel proportion (V_-) was defined to capture the global spatial extent of downregulation, and the composite cholinergic progression measure (C) was used to capture the joint process of loss in hyper-cholinergic voxels and gain in hypo-cholinergic voxels that occurs with disease progression.

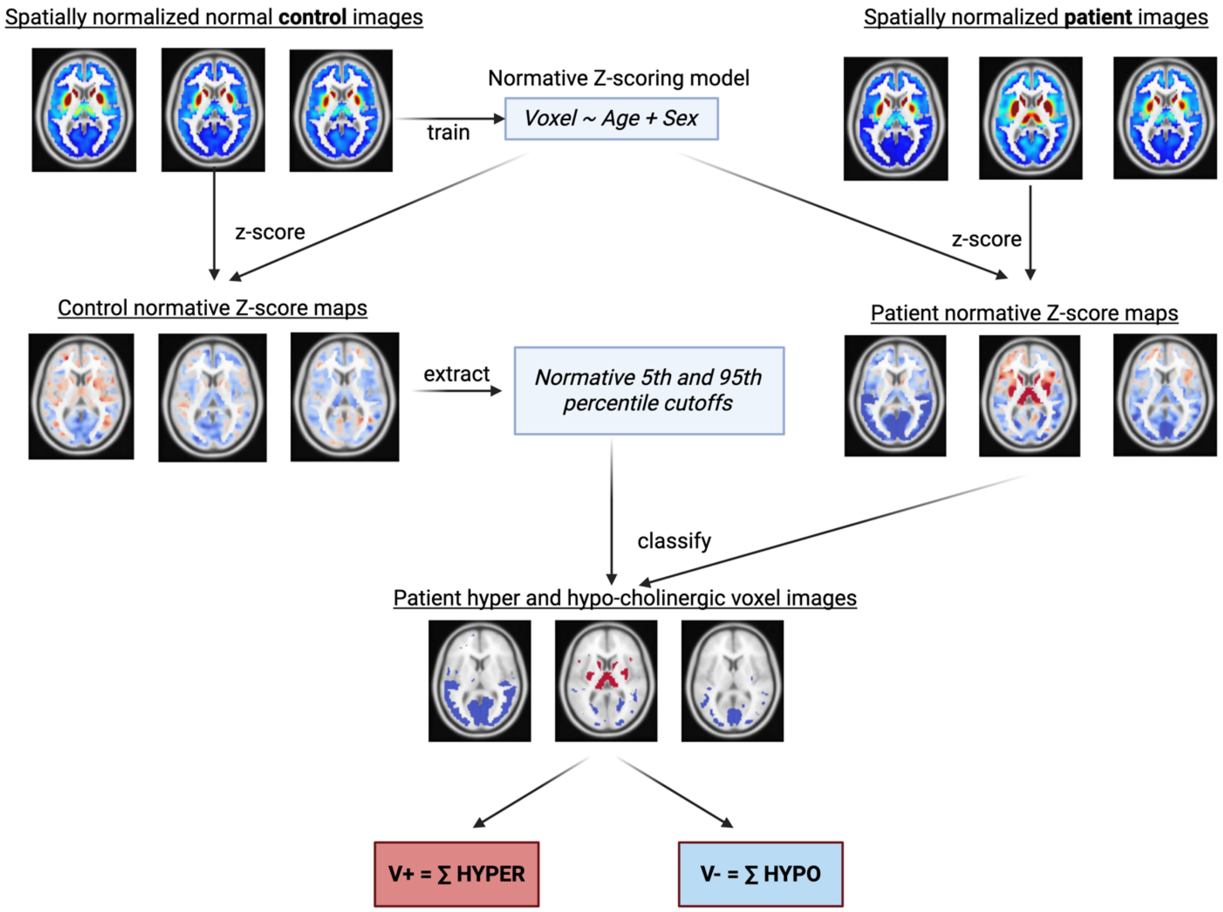

Fig. S10. Schematic representation of the algorithm used to obtain hypo and hyper-cholinergic voxel counts. Spatially normalized [^{18}F]FEOBV PET parametric images of normal controls were used to fit a voxelwise linear regression model, predicting each voxel value from a set of pre-defined confounder variables. The resulting voxelwise linear regression models were used to convert every voxel raw value (for both controls and patients) to a normative Z-score as described in methods subhead 2 (blue: lower than normative, red: higher than normative). The sample of controls was used to define 5th and 95th percentile cutoffs corresponding to thresholds

for being classified either hypo (<5th percentile of controls) or hyper (>95th percentile of controls). For each patient, all the image voxels were classified as either hyper (red voxels), hypo (blue voxels), or neither (no color). Finally, the number of hyper and hypo voxels within each patient's image is counted and used to derive the global summary statistics as described in methods subhead 6. Created in BioRender. Roytman, S. (2025) https://BioRender.com/ofpuiuo.

The three global measures were plotted over age (as a rough proxy of disease timeline) in the baseline sample of controls, which demonstrate the progression of these processes (figure S11).

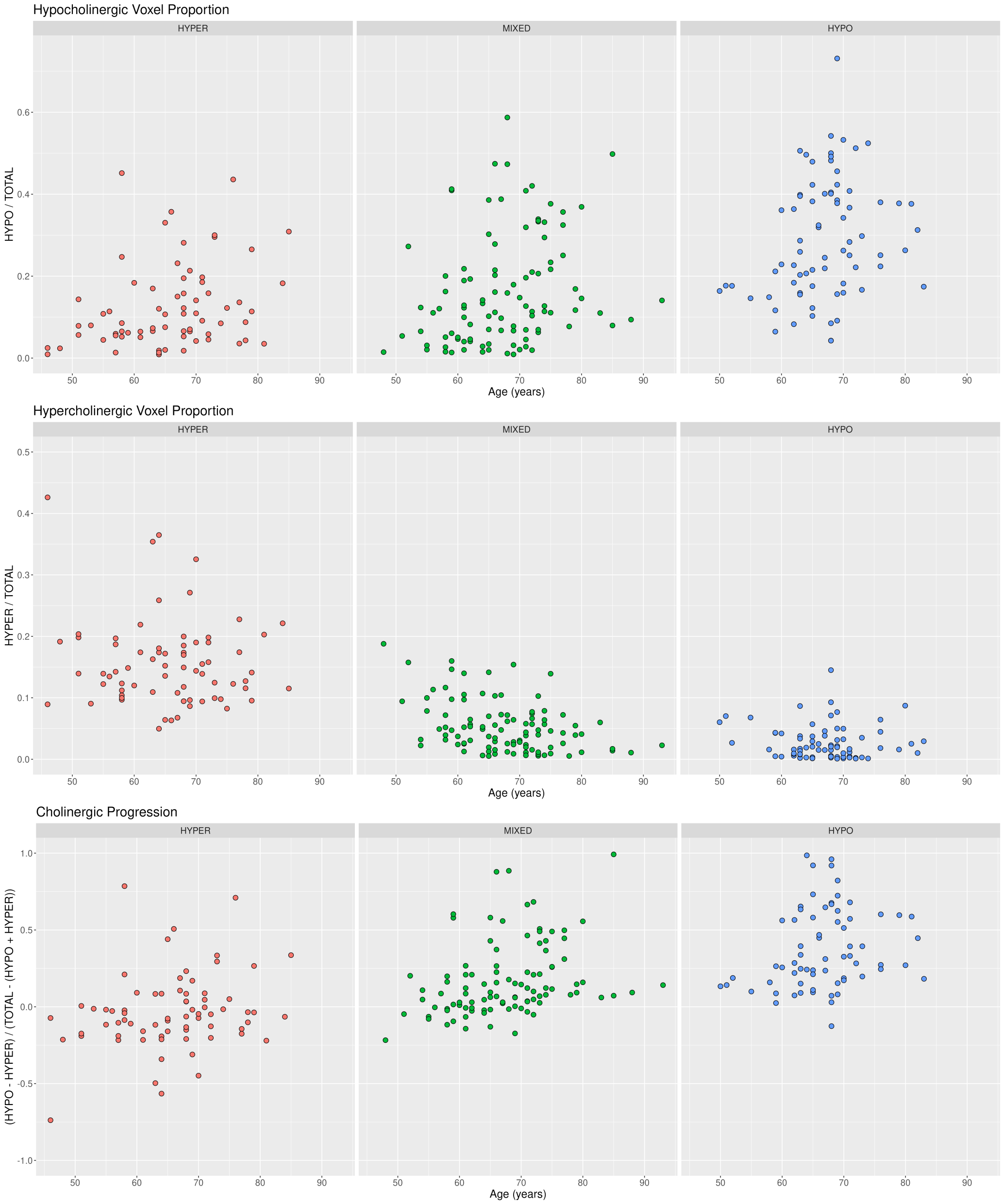

Fig. S11. Global cholinergic system summary measures plotted over age by cholinergic subgroup. Red color indicates hyper-cholinergic subgroup, green indicates mixed-cholinergic subgroup, blue indicates hypo-cholinergic subgroup.

In order to verify that the resulting global cholinergic system measures are closely related to the principal components used to define the subgroups, a set of multivariate regression models were fitted predicting each measure from the five principal component scores among PD patients at baseline (table S3). Every global measure is strongly predicted by the set of principal components based on adjusted coefficients of determination (R^2), and the pattern of strongly predictive principal components on each measure are consistent with their interpretation. V_+ is most strongly predicted by the cerebellar principal component (PC4), followed by the centro-cingulate (PC2) and entorhinal (PC5) principal components, in all of which some extent of upregulation is observed on either the primary voxelwise group comparison against controls or the supplementary analyses by subgroups with and without PVC presented in supplementary materials section 7. Notably, the limbic/subcortical component (PC3) is not independently predictive of the V_+, which may be explained by the strong overlap in variance that it has with the cerebellar principal component (r=0.73 [0.64, 0.82], p<0.001, R^2=0.531). The strongest independent predictor of V_- is the posterior cortical component (PC1), followed by entorhinal (PC5) and subcortical components (PC3), which is consistent with most prominent loss of cholinergic innervation relative to controls being observed in posterior cortices across all the subgroup, and more severe cholinergic denervation of subcortical regions and medial temporal structures observed specifically in the hypo-cholinergic subgroup. Lastly, strongest independent predictors of C are the posterior cortical and entorhinal components, both of which figure prominently as predictors of V_-. This is consistent with a multivariate model predicting C from the other two global measures (R^2=0.916), which demonstrates that overall cholinergic progression is more prominently driven by the gain of hypo-cholinergic voxels (β=0.85 [0.81, 0.89], p<0.001) than by the loss of hypo-cholinergic voxels (β=-0.22 [-0.26, -0.18], p<0.001). Taken together, these findings show that close correspondence between global cholinergic system summary measures and the system-level principal components used to define the subgroups.

Table S4. Multivariate model of global cholinergic system summary measures predicted from principal component scores used to define the subgroups on cross-sectional baseline data. V_+ represents the hyper-cholinergic (above 95th percentile of controls) voxel proportion, V_- represents the hypo-cholinergic voxel proportion (below 5th percentile of controls), and C represents the global cholinergic system progression (a composite of the prior two). Strong regression coefficients wherein the confidence interval does not overlap with 0 are marked with an asterisk.

Variable	*PC1*	*PC2*	*PC3*	*PC4*	*PC5*	R^2
V_+	+0.09 [-0.07, 0.25]	+0.24* [0.03, 0.44]	+0.09 [-0.06, 0.23]	+0.48* [0.37, 0.59]	+0.1* [0, 0.2]	0.685
V_-	-0.79* [-0.98, -0.59]	+0.21 [-0.03, 0.45]	+0.19* [0.01, 0.36]	+0.03 [-0.1, 0.16]	-0.34* [-0.46, -0.22]	0.559
C	-0.58* [-0.78, -0.38]	+0.03 [-0.21, 0.28]	+0.14 [-0.04, 0.32]	-0.09 [-0.23, 0.04]	-0.31* [-0.43, -0.19]	0.540

To demonstrate that the behavior of these global summary measures agrees with the observation of longitudinal changes by subgroup observed on the voxelwise comparison, a set of

mixed linear models were fitted, predicting each global measure from visit by subgroup interaction while controlling for interval duration between visits. Estimated marginal means with 95% confidence intervals were obtained to characterize the distribution of these measures in the combined sample at baseline and within-subgroup follow-up minus baseline contrast was obtained to characterize the longitudinal changes by subgroup.

V_+ was estimated at 15.3% (CI_{95}=[14.2, 16.4]%) of brain voxels at baseline for the hyper-cholinergic subgroup, who exhibited the strongest decrease after a ~2.5 year interval of 3.4% of total voxels (t_{ratio}=-5.898, p<0.001). In mixed- and hypo-cholinergic subgroups V_+ at baseline was substantially diminished, at 5.4% (CI_{95}=[4.4, 6.3]%) and 2.9% (CI_{95}=[1.8, 4.0]%) of total brain voxels respectively, with neither subgroup exhibiting a substantial further reduction in V_+ longitudinally.

V_- was estimated at 11.5% (CI_{95}=[8.4, 14.7]%) of total brain voxels at baseline for the hyper-cholinergic subgroup, who exhibited the strongest interval increase of 6.8% of total voxels (t_{ratio}=5.722, p<0.001). In mixed-cholinergic subgroups V_- was somewhat elevated, at 16.5% (CI_{95}=[13.9, 19.1]%) of total brain voxels, and still saw a significant interval increase at 5.1% of total brain voxels (t_{ratio}=5.152, p<0.001). While the hypo-cholinergic subgroup exhibited the greatest baseline V_- at 29.7% (CI_{95}=[26.6, 32.7]%) of total brain voxels, their interval increase at 2% of total brain voxels fell short of significance (t_{ratio}=1.689, p=0.0935).

C, which measures the relative predominance of hypo-cholinergic over hyper-cholinergic brain voxels was lowest in the hyper-cholinergic subgroup at baseline, trending towards relative predominance of hyper-cholinergic voxels (C=-0.043 [-0.119, 0.034]) and shifted towards relative predominance of hypo-cholinergic voxels at the greatest rate of the three subgroups longitudinally (ΔC=0.16, t_{ratio}=6.174, p<0.001). The mixed-cholinergic subgroup already exhibited relative predominance of hypo-cholinergic brain voxels at baseline (C=0.18 [0.12, 0.25]), but longitudinally progressed at a slower rate however than the hyper-cholinergic subgroup (ΔC=0.098, t_{ratio}=4.454, p<0.001). Lastly, while the hypo-cholinergic subgroup demonstrated the greatest predominance of hypo-cholinergic voxels of the three subgroups at baseline (C=0.47 [0.4, 0.55]), the interval change in C was the weakest and no longer statistically significant (ΔC=0.038, t_{ratio}=1.395, p=0.165).

In summary, the analysis of longitudinal interval changes in global cholinergic system summary metrics support the same conclusion implied by our voxelwise paired t-test comparison by baseline subgroup – the progression of cholinergic system changes towards the hypo-cholinergic state occurs at the greatest rate in the hyper-cholinergic subgroup and decelerates progressively as individuals transition to mixed- and hypo-cholinergic subgroups. Taken together with multivariate models predicting summary measures cross-sectionally, these supplementary analyses demonstrate these global summary metrics align well with both the systems-level description of the cholinergic system provided by principal component scores, and the voxel-level dynamics of cholinergic innervation interval changes observed in the paired samples t-test within subject comparison.

References

1. P. Kanel, S. van der Zee, C. A. Sanchez-Catasus, R. A. Koeppe, P. J. H. Scott, T. van Laar, R. L. Albin, N. I. Bohnen, Cerebral topography of vesicular cholinergic transporter changes in neurologically intact adults: A [18F]FEOBV PET study. *Aging Brain* **2**, 100039 (2022).

2. X. Shao, R. Hoareau, B. G. Hockley, L. J. M. Tluczek, B. D. Henderson, H. C. Padgett, P. J. H. Scott, Highlighting the versatility of the Tracerlab synthesis modules. Part 1: Fully automated production of [F]labelled radiopharmaceuticals using a Tracerlab FX(FN). *J. Labelled Comp. Radiopharm.* **54**, 292–307 (2011).

3. X. Shao, R. Hoareau, A. C. Runkle, L. J. M. Tluczek, B. G. Hockley, B. D. Henderson, P. J. H. Scott, Highlighting the versatility of the Tracerlab synthesis modules. Part 2: fully automated production of [11C]-labeled radiopharmaceuticals using a Tracerlab FXC-Pro. *J. Labelled Comp. Radiopharm.* **54**, 819–838 (2011).

4. M. Petrou, K. A. Frey, M. R. Kilbourn, P. J. H. Scott, D. M. Raffel, N. I. Bohnen, M. L. T. M. Müller, R. L. Albin, R. A. Koeppe, In vivo imaging of human cholinergic nerve terminals with (-)-5-(18)F-fluoroethoxybenzovesamicol: biodistribution, dosimetry, and tracer kinetic analyses. *J. Nucl. Med.* **55**, 396–404 (2014).

5. S. Minoshima, R. A. Koeppe, J. A. Fessler, M. A. Mintun, K. L. Berger,). Stephan F. Taylor, D. E. Kuhl), Integrated and automated data analysis method for neuronal activation studies using 0-15 water PET. https://web.eecs.umich.edu/~fessler/papers/files/abs/9x/minoshima-93-iaa-1.pdf.

6. M. Aghourian, C. Legault-Denis, J.-P. Soucy, P. Rosa-Neto, S. Gauthier, A. Kostikov, P. Gravel, M.-A. Bédard, Quantification of brain cholinergic denervation in Alzheimer's disease using PET imaging with [18F]-FEOBV. *Mol. Psychiatry* **22**, 1531–1538 (2017).

7. S. Nejad-Davarani, R. A. Koeppe, R. L. Albin, K. A. Frey, M. L. T. M. Müller, N. I. Bohnen, Quantification of brain cholinergic denervation in dementia with Lewy bodies using PET imaging with [18F]-FEOBV, *Molecular psychiatry*. **24** (2019)pp. 322–327.

8. H. W. Müller-Gärtner, J. M. Links, J. L. Prince, R. N. Bryan, E. McVeigh, J. P. Leal, C. Davatzikos, J. J. Frost, Measurement of radiotracer concentration in brain gray matter using positron emission tomography: MRI-based correction for partial volume effects. *J. Cereb. Blood Flow Metab.* **12**, 571–583 (1992).

9. V. M. Bashyam, G. Erus, J. Doshi, M. Habes, I. Nasrallah, M. Truelove-Hill, D. Srinivasan, L. Mamourian, R. Pomponio, Y. Fan, L. J. Launer, C. L. Masters, P. Maruff, C. Zhuo, H. Völzke, S. C. Johnson, J. Fripp, N. Koutsouleris, T. D. Satterthwaite, D. Wolf, R. E. Gur, R. C. Gur, J. Morris, M. S. Albert, H. J. Grabe, S. Resnick, R. N. Bryan, D. A. Wolk, H. Shou, C. Davatzikos, MRI signatures of brain age and disease over the lifespan based on a deep brain network and 14 468 individuals worldwide. *Brain* **143**, 2312–2324 (2020).
